# Recent advances in patient and proxy-reported quality of life research

**DOI:** 10.1186/s12955-014-0110-7

**Published:** 2014-08-29

**Authors:** Kristofer Bandayrel, Bradley C Johnston

**Affiliations:** Child Health Evaluative Sciences, The Hospital for Sick Children Research Institute, Toronto, Ontario Canada; Department of Anesthesia and Pain Medicine, Hospital for Sick Children, Toronto, Ontario Canada; Institute of Health Policy, Management and Evaluation, University of Toronto, Toronto, Ontario Canada; Peter Gilgan Centre for Research and Learning, Hospital for Sick Children Research Institute, 686 Bay Street, Room 11.9848, Toronto, ON M5G 0A4 Canada

## Abstract

**Background:**

A number of articles addressing various aspects of health-related quality of life (HRQoL) were published in the Health and Quality of Life Outcomes (HQLO) journal in 2012 and 2013. This review provides a summary of studies describing recent methodological advances and innovations in HRQoL felt to be of relevance to clinicians and researchers.

**Methods:**

Scoping review of original research articles, reviews and short reports published in the HQLO journal in 2012 and 2013. Publications describing methodological advances and innovations in HRQoL were reviewed in detail, summarized and grouped into thematic categories.

**Results:**

358 titles and abstracts were screened initially, and 16 were considered relevant and incorporated in this review. Two studies discussed development and interpretation of HRQoL outcomes; two described pediatric HRQoL measurement; four involved incorporation of HRQoL in economic evaluations; and eight described methodological issues and innovations in HRQoL measures.

**Conclusions:**

Several studies describing important advancements and innovations in HRQoL, such as the development of the PROMIS pediatric proxy-item bank and guidelines for constructing patient-reported outcome (PRO) instruments, were published in the HQLO journal in 2012 and 2013. Proposed future directions for the majority of these studies include extension and further validation of the research across a diverse range of health conditions.

## Introduction

Over 350 research articles, reviews and short reports were published in the Health and Quality of Life Outcomes (HQLO) journal in 2012 and 2013. Collectively these publications addressed a broad range of topics in health-related quality of life (HRQoL) such as alternative approaches for presenting pooled estimates of patient-reported outcomes (PROs); parent-proxy reporting and the Patient-Reported Outcomes Measurement Information System (PROMIS) pediatric proxy item bank; mapping disease-specific instrument scores onto generic measures; and issues related to evaluating health status changes in various health conditions. This scoping review aims to provide a summary of the key advances from the HQLO 2012 and 2013 publications felt to be relevant to researchers and clinicians. One reviewer (KB) initially screened all the titles and the abstracts of the 2012 and 2013 HQLO publications, and discussed potentially relevant studies with a second reviewer (BCJ). The full-text publications were then assessed, and those still considered relevant were summarized and grouped into one of four categories discussed in detail below: (1) development and interpretation of HRQoL outcomes; (2) pediatric HRQoL measurement; (3) incorporation of HRQoL in economic evaluations; and (4) methodological issues and innovations in HRQoL meaures.

### Development and interpretation of HRQoL outcomes

Conceptual models improve our understanding of a complex phenomenon such as HRQoL by providing a schematic representation of a theory and portraying the inter-relationships between concepts [1]. Differences in terminology for analogous HRQoL concepts, however, have made comparisons across studies challenging and limited the capacity to develop a rigorous body of evidence to guide future HRQoL research and practice [2]. To advance the conceptualization of HRQoL using a common language, Bakas et al. [2] performed a systematic review to identify and assess the most frequently applied HRQoL models over the past ten years. Though their findings revealed little consensus in the use of HRQoL models between studies, among those commonly applied were the Wilson and Cleary model of HRQoL, [3] Ferrans and colleagues’ revision of the Wilson and Cleary model [4] and the World Health Organization International Classification of Functioning, Disability and Health (WHO ICF) [5]. Wilson and Cleary’s model combines biomedical and social science paradigms, and consists of 5 related domains: biological, symptoms, function, general health perception and overall HRQoL. Ferrans and colleagues’ revision enhances this model by retaining these domains and adding individual and environmental characteristics [4]. The WHO ICF model provides a standard language for health and health states applicable across disciplines and cultures, and includes functioning and disability components (e.g. body functioning, participation) and contextual (environmental and personal) factors. A critical analysis of the models using Bredow’s criteria [6] showed that all three were complete in their descriptions and definitions of HRQoL, and applicable to real-world settings. The Ferrans and colleagues’ model, however, provided the added benefit of clarity in conceptual and operational definitions and relationships among concepts. As such, the authors recommended the use of the Ferran’s model to improve comparisons of HRQoL between studies and facilitate the development of a robust body of evidence for future HRQoL research and practice.

HRQoL is often measured as a patient-reported outcome (PRO), described as “any report of the patient’s health condition that comes directly from the patient without interpretation of the patient’s response by a clinician or anyone else” [7]. As clinical trials continue to incorporate PROs to measure outcomes beyond morbidity and mortality, systematic reviews and meta-analyses authors contend with the challenge of presenting pooled PRO estimates. When pooling across different HRQoL instruments that measure a common construct, the weighted mean difference is much more challenging to generate and is replaced with a unitless measure of effect called the standardized mean difference (SMD). The publication by Johnston et al. [8] provides an overview of 5 summary approaches for enhancing the interpretability of pooled PRO estimates: (1) standardized mean difference (difference in means in each trial divided by the estimated between-person standard deviation) (2) natural units (linear transformation of trial data to most familiar scale) (3) relative and absolute dichotomized effects (proportion above a pre-determined threshold presented as a binary effect measure) (4) ratio of means (ratio between the mean responses in the intervention and control group), and (5) minimal important difference (MID) units (pooled mean difference presented in MID units, where instead of dividing the mean difference of each study by its standard deviation, this method divides by the MID associated with the PRO measures). When trials all use the same PRO it is important to report results beyond a mean difference and statistical significance. When primary studies have employed more than one instrument it will almost certainly be informative to report one or more alternatives to the SMD. Calculation and reporting of several approaches will be reassuring, provided the estimate of effect is of apparently similar magnitude; if not, this presents a challenge that reviewers should address.

### Pediatric HRQoL measurement

PROMIS was initiated by the National Institutes of Health (NIH) in 2004 and was aimed at providing clinicians and researchers with important PRO information not captured by clinical measures, and could also be used as endpoints in clinical studies evaluating the effectiveness of treatments for chronic health conditions [9]. This was achieved by (1) establishing a domain framework, defined as the structure of a target domain such as physical health (2) determining the conceptual framework or hierarchical structure of the domain (3) developing and validating items that could be grouped into a set of item banks. The PROMIS pediatric project focused on developing self-reported PRO item banks among those aged 8 to 17 years, with a focus on the measurement of general health domains felt to be important across various health states [10] as well as an additional disease-specific item bank specifically for children with asthma [11]. In 2010, additional item banks were developed, and longitudinal validation studies were conducted in new populations and for new treatment [9]. A pediatric proxy item bank was developed for those age 5 to 17 years as part of this initiative, and to address the need for health status instruments reflecting the perspectives of both the child and parent in cases where a child is too young, cognitively impaired or unwell to complete a PRO instrument and a parent proxy report is required [12]. Though proxy responses are often not equivalent to those provided directly by a patient, [13–16] it is typically the parents’ perception of their child’s symptoms and outcomes that influence healthcare utilization [12]. For these reasons, Irwin et al. [10] developed an initial PROMIS pediatric proxy-report item bank, [17] consisting of the following five health domains: physical function; emotional distress; social peer relationships; pain interference; and asthma impact. The authors acknowledge that further research is needed to establish construct validity and responsiveness in larger samples of caregivers of children with chronic health conditions [10].

As mentioned, parent-proxy report can often be a limitation in the assessment of HRQoL, [18] with only a few studies evaluating the level of agreement between parents and children on a child’s HRQoL over time. To explore this issue further Rajmil et al. [19] conducted a 3-year sub-study of the European Screening for and promotion of HRQoL in children and adolescents (KIDSCREEN) project. The primary focus of the study was to explore the association between age and time of follow-up on the level of agreement, as measured by the KIDSCREEN-27 [20] and KIDSCREEN-10 [21] questionnaires, between parent and child on the child’s HRQoL. The analysis showed low to moderate levels of parent–child agreement at baseline and lower agreement at follow-up; child’s age and parent’s self-perceived health were the primary factors associated with parent–child disagreements over time. Based on these findings Rajmil et al. recommended direct self-assessment of HRQoL among children and adolescents as much as possible, and acknowledge that their results may have been biased by factors such as low response rates (54 %) and the generally healthy characteristics of their study sample.

### Incorporation of HRQoL in economic evaluations

In selecting an instrument to measure quality of life (QoL), its impact on the resulting cost**-**effectiveness of a medical intervention should be considered as cost**-**effectiveness is often determined as a cost per quality-adjusted life year (QALY), a measure combining length of time with quality of life [22]. Disease-specific instruments are often preferred over generic ones when measuring QoL as these tend to focus on specific health problems and are more sensitive to clinically important differences [23]. Generic measures such as the EuroQOL 5-Dimension scale (EQ-5D), however, provide a single preference‐based score that is required for cost‐utility analysis and calculation of QALYs. One proposed solution to this issue is to “map” disease‐specific measures onto generic ones using regression analysis to establish the relationship between preference-based indices and the dimension or item scores of disease‐specific measures, thereby obtaining estimation models that can be used to calculate QALYs [24,25]. Although a mapping relationship between the European Organization for Research and Treatment of Cancer Quality of Life Questionnaire Core 30 (EORTC QLQ C30) and the utility based values of the EQ 5D had been previously established, the sample used to derive these estimates consisted of patients with a single type of cancer. Kim et al. [26] aimed to extend this work to patients with a wide range of cancers in Korea. The results of the final mapping model demonstrated reasonable predictive ability, and the authors suggested that the resulting mapping algorithm could potentially inform future cost utility analysis of healthcare interventions by converting the results of the EORTC QLQ 30 to ED 5D utility indices.

Dakin et al. [27] expanded this work by conducting a structured literature review aimed at identifying studies mapping to the EQ-5D. 90 studies reporting 121 mapping algorithms had met the study inclusion criteria, of which 22 involved indirect mapping, and 28 corresponded to musculoskeletal disease. Dakin notes that the majority of studies were from 2009 to 2012, which can perhaps be attributed to the publication of the 2008 NICE methods guide for mapping in the absence of directly measured EQ-5D [28] and guidance document on mapping methodology [29]. The publicly available database of mapping studies is available through: http://www.herc.ox.ac.uk/downloads/mappingdatabase. Though this database provides researchers with a resource for identify mapping algorithms linking various instruments with the EQ-5D, Dakin cautions that no quality assessment was performed on any of the included studies, and that mapping should always be considered secondary to direct EQ-5D measurement, as mapping may introduce additional errors and assumptions.

Both disease‐specific and generic health status instruments can provide important and at times complimentary insights into the HRQoL of patients affected by chronic disease and inform the cost effectiveness of different healthcare interventions [30]. Wilke et al. [31] carried out a one-year, observational study of patients with advanced chronic obstructive pulmonary disease to determine whether and to what extent the scores from a disease specific questionnaire, the St. George Respiratory Questionnaire (SGRQ), correlate with generic health status instruments over time, specifically the EQ-5D; Medical Outcomes Study 36‐item Short Form Survey (SF-36) Physical Component Summary Measure (PCS) and Mental Component Summary Measure (MCS); and the Assessment of Quality of Life (AQoL) instrument. Patients completed each of these questionnaires at four time points (baseline, 4, 8 and 12 months), and the following thresholds used to classify the strength of the correlation: absent (<±0.20); weak (±0.20 to ±0.34); moderate (±0.35 to ±0.50); and strong (> ± 0.50) [32]. Correlations between the SGRQ total score and the scores from each of the generic instruments ranged from weak to strong at the four time points. At baseline, the disease-specific and generic health status questionnaires were moderately to strongly correlated, though over time the correlations between the changes were weak or absent.

Given the increasing need to use appropriate outcome measures in health economics research, [33–35] Jones et al. [22] performed a systematic review to identify the outcome measures most frequently used in health interventions involving caregivers of patients with dementia, and the usefulness of these measures for economic evaluation. To be considered for inclusion, studies had to report an intervention with outcome measures for care providers of persons with dementia such as paid workers or informal caregivers (e.g. family or friends). Outcomes for paid workers were included to achieve a broader indication of which aspects of health and social care provision are typically measured. Their search identified 455 articles reporting on 361 studies. Twenty‐nine studies included details of costs, of which the majority were only partial economic evaluations that provided cost‐outcome descriptions (e.g. cost per additional year that the person with dementia lived at home). Three studies [36–38] included a cost‐utility analysis using three generic health measures suitable for QALY calculations: the EQ‐5D [36], Health Utility Index-2 (HUI2) [37] and the Caregiver Quality of Life Instrument [38]. Since the decision to use a specific QoL measure has implications on its cost-effectiveness, the authors suggest that health economists select instruments appropriate to their intended population and outcomes of interest, and that clinical trialists consider ease of administration, time constraints, clarity and respondent burden when choosing an appropriate measure.

### Methodological issues and innovations in HRQoL measures

Potential sources of bias when evaluating patient‐reported outcomes (PROs) include the lack of measurement equivalence, selection bias and the methods and instruments used to evaluate changes in health status.

*Measurement equivalence* refers to the perception that individuals from different populations will interpret a measurement (e.g. PROs) in a conceptually similar manner [39,40]. In cases where an instrument lacks this property, for instance when study participants may have different frames of references to respond to questions about their health, [41,42] between‐group differences may be confounded by measurement artifact and thus not reflect true differences in the population. Given the frequent use of the SF‐36 in over 50 countries [43] and the lack of studies evaluating its measurement equivalence properties, Lix et al. [39] assessed its measurement equivalence by sex and race using data from the Canadian Multi-centre Osteoporosis Study (CaMos) [44]. In brief, CaMos was a prospective cohort study that aimed to assess the burden, including the health and economic consequences, of osteoporosis and fracture among Canadian women and men and identify factors associated with these conditions [45]. Participants were aged 25 years or older, community‐dwelling, and living within a 50-kilometer radius of a study site [44]. The results of the confirmatory factor analysis revealed that all forms of measurement equivalence were satisfied for each of the four groups in this study: Caucasian and non-Caucasian females; Caucasian and non‐Caucasian males; Caucasian males and females; and non‐Caucasian males. The study results further demonstrated that sex and race did not influence the conceptualization of a general measure of HRQoL among participants enrolled in the CaMos study [39].

*Selection bias* due to non‐response is another issue when assessing PRO measures, as prior studies have shown that non-responders have generally poorer health outcomes when compared to responders [46–50]. In a study assessing non‐response rates to post‐operative questionnaires and patient characteristics among National Health Service (NHS) hospitals in England, Hutchings et al. [51] found that non-response was significantly associated with socio‐demographic and clinical characteristics, specifically: male gender, younger age, low socio‐economic status and relatively poor pre‐operative health. The authors emphasize that the implication of their findings depend on the extent to which non‐response is associated with outcomes, though it is not quite clear whether this applies to similar observational studies, randomized trials, or both.

Coste et al. [52] conducted a similar study assessing the patterns, determinants and impact of non- (missing forms), incomplete (missing items) and inconsistent (occurrence of inconsistency between items) responses on the validity of HRQoL estimates, as measured by the SF-36, among a representative sample of French adults participating in the 2003 Decennial Health Survey (n = 30,782). Several factors were associated with non and partial responses, of which the strongest were educational level (lower educational level) and age (18–25 years or > 50 years); other factors included: occupation (being economically active), foreign background, low income (females only), region of residence (males only), being single, divorced or widowed (males and females) and morbidity. To evaluate the impact of non and partial responses on the validity of the HRQoL estimates, multiple imputation methods were applied to provide the best-corrected estimates against which the magnitude of the biases were assessed. This analysis indicated that the magnitude of the biases were large among non-responders and several groups of partial responders, and confirmed a “missing, not-random” process of missing information in HRQoL measurement [28]. Consequently, the authors strongly recommend the use of missing value methods, such as multiple imputation, to systematically evaluate the consequences of missing and partial responses on HRQoL estimations [29,53,54].

*Evaluating changes in health status* can also be a challenging task, as controversy exists regarding the best method for determining baseline health status. In studies evaluating change in health status for an acute‐onset condition such as an injury (e.g. fracture, sprain or concussion), pre‐injury health status is often determined in one of two ways following the event: retrospective evaluation of pre‐injury health, or use of population norms as a proxy measure for pre-injury health [55]. Wilson et al. [55] assessed the validity of these two approaches using EQ-5D data from the Prospective Outcomes of Injury study (POIS). In this study, participants were asked to recall their pre-injury (baseline) health at 3 months following the injury, and their current health at 5 and 12 months follow-up. Participants were further classified as fully recovered or non-recovered based on a self-assessment of their recovery status at follow-up, and their scores on the World Health Organization Disability Assessment Schedule (WHODAS 2.0), an instrument developed by the World Health Organization (WHO) used to measure disability [56]. The authors hypothesized that if recalled pre-injury health valuations were unbiased, then (1) pre-injury health state values would be statistically similar to post-injury values among those fully recovered, and (2) pre-injury health state values would be significantly higher than post-injury values for those who were non-recovered. Likewise, if population norms were a valid proxy for pre-injury health then population norms would approximate the health status of participants who were fully recovered. Their analysis showed a small, albeit statistically significant, positive difference for participants who had fully recovered, and a large positive difference among those not fully recovered; these differences remained at the two follow-up time points. In comparing the EQ-5D data with the general population, both recovered and non-recovered participants reported significantly better pre-injury health than the population norm. At both follow-up time points reported health among those who were fully recovered remained higher than the general population, while those who were non-recovered were significantly lower. These findings showed that both retrospectively measured pre-injury health status and population norms differed from those fully recovered from injury. Based on the magnitude of the differences, Wilson et al. support the use of retrospective evaluation as these estimates were found to be more precise, though they caution that there may be a small upward bias with this approach.

The use of different instruments to assess HRQoL for a given health condition could potentially result in non-comparable estimates, which in turn may have an impact on the cost-effectiveness and health utility of an intervention. This has led some to suggest that for certain health conditions, one specific instrument to measure HRQoL may be more appropriate to use than others. Turner et al. [57] evaluated the agreement between, and suitability of, four different instruments for measuring health utility in depressed patients: (1) EQ-5D-3 L; (2) EQ-5D Visual Analog Scale (EQ-5D VAS); (3) SF-6D; and (4) SF-12 new algorithm. Their findings indicated a low level of agreement between the four instruments (overall intra-class correlation (ICC) of 0.57), though Bland and Altman plots provided evidence that the SF-6D and SF-12 new algorithm instruments could be used interchangeably. Plots of the health utility score from each of the instruments against one another displayed ceiling and floor effects in the EQ-5D-3 L index scores and SF-6D and SF-12 new algorithm, respectively, though all instruments demonstrated responsiveness to change and had relatively high completion rates. Based on their results the authors suggest that the SF-12 new algorithm may be more appropriate for measuring HRQoL than the EQ-5D-3 L.

Similarly, Kuspinar et al. [58] assessed the extent to which common generic utility measures such as the Health Utility Index-2 (HUI2), Health Utility Index-3 (HUI3), EQ-5D and SF-6D capture important and relevant domains for persons with multiple sclerosis (MS), as missing important domains could contribute to biased cost-effectiveness analyses due to invalid comparisons across interventions and populations resulting in inaccurate QALYs. Of the top 10 domains that the study sample (n = 185) identified to be most affected by their MS (work, fatigue, sports, social life, relationships, walking, cognition, balance, housework and mood), none of the generic instruments were found to be comprehensive: the SF-6D captured 6 domains, followed by the EQ-5D (4 domains), HUI2 (4 domains) and HUI3 (3 domains). Furthermore, the generic utility measures included several domains such as pain, self-care, vision, hearing, manual dexterity, speech and fertility that were not identified as important by the study sample. Though imprecise, the authors suggest that the use of the SF-6D may be the most appropriate to use among persons with MS compared to other generic utility measures, and further propose the development of MS specific “bolt-on” items to generic utility measures [59], or an MS-specific utility measure consisting of only disease-specific dimensions.

The term rating scales refers to the response options within a PRO instrument, and are commonly presented as a set of categories defined by descriptive labels [60]. In the absence of high quality evidence or general consensus on optimal methods, PRO developers may take various approaches in constructing a rating scale such as the use of verbal descriptors to express attitudes (e.g. strongly disagree, disagree, agree, strongly agree). In developing these scales certain trade-offs must be taken into account such as achieving finer discrimination through more response categories versus respondent burden and capacity to discern between categories, though there is a lack of clear guidelines to inform this decision. Khadka et al. [61] aimed to explore the characteristics of functional and dysfunctional rating scales, and in doing so develop evidence-based guidelines for constructing rating scales. Their study sample consisted of adults age 18 years or older who were on a cataract surgical waiting list in South Australia. All participants were asked to complete a package of 10 self-administered PRO measures (rotationally selected from a pool of 17 PRO instruments used to measure the impact of cataract surgery). Each of the 17 measures assessed various vision-related QoL dimensions using ratings from four concepts: difficulty (e.g. reading small print); frequency (e.g. times worrying about worsening eyesight in past month); severity (e.g. pain or discomfort in and around eyes); and global ratings (e.g. global rating of vision). Based on the results of the Rasch analysis, a probabilistic mathematical model that estimates interval measures from ordinal raw data and provides a strong assessment of rating scale function [62], Khadka et al. found that items with simple and uniform question formats and four or five labeled categories were most likely to be functional and often demonstrated hierarchical ordering and good coverage of the latent trait under measurement [61]. In contrast, PRO measures with a larger number of categories and complicated question formats were likely to have a dysfunctional rating scale. While a brief summary of the guidelines for developing rating scales is provided, Khadka et al. emphasize the continuing need to exercise sound judgment, on the basis of the construct being measured and research question, when developing a rating scale. The authors further acknowledge that their study was limited to PRO measures specific to ophthalmology, though they note that their work may have broader relevance and call for its replication in other disciplines.

Krabbe and Forkman [63] proposed to determine whether frequency or intensity scales should be employed as verbal anchors in self-report instruments among patients with a depressive disorder. Verbal anchors refer to terms used within a set of statements of a self-report instrument indicating the frequency (e.g. never, sometimes, always) or intensity (e.g. not at all, moderately, extremely) of the symptoms associated with a specific health condition [63]. The authors applied three criteria to compare the appropriateness of using either frequency or intensity terms: inter-individual congruency of mental representations of terms; intra-individual stability across time of mental representations of terms; and distinguishability of adjacent terms. The authors found that both scales could be applied as verbal anchors, though they cautioned against using more than four adjacent terms in a rating scale, as patients with a depressive disorder may not be able to reasonably distinguish more than four. They further suggest the use of frequency-related terms if longitudinal assessment is required, as this study provided preliminary evidence that terms pertaining to frequency had slightly higher intra-individual stability over time compared to those referring to intensity [63].

## Conclusion

This scoping review provides a summary of original research articles, reviews and short reports describing methodological advancements and innovations in QoL and HRQoL felt to be of significance to clinicians and researchers and published in the HQLO journal in 2012 and 2013. Of 358 publications, 16 were considered relevant, summarized and grouped into thematic categories (Table 1).

**Table 1:** **Summary of key issues and corresponding HQLO references**

**Key issue**	**Author, year [reference number]**
Development & interpretation of HRQoL outcomes	Bakas T et al., 2012 [2]; Johnston BC et al., 2013 [8]
Pediatric HRQoL measurement	Irwin DE et al., 2012 [10]; Rajmil L et al., 2013 [19]
Incorporation of HRQoL in economic evaluations	Jones C et al., 2012 [22]; Kim SH et al., 2012 [26]; Dakin H, 2013 [27]; Wilke S et al., 2012 [31]
Methodological issues and innovations in HRQoL measures	
*Measurement equivalence*	Lix M et al., 2012 [42];
*Selection bias*	Hutchings A et al., 2012 [54]; Coste J et al., 2013 [55]
*Evaluating change in health status*	Wilson R et al., 2012 [58]; Turner N et al., 2013 [60]; Kuspinar A et al., 2013 [61]; Khadka J et al., 2012 [61]; Krabbe J et al., 2012 [63]

In summary, two studies were relevant to the development and interpretation of HRQoL outcomes. The literature review by Bakas et al. [2] found little consensus in the types of HRQoL models used between studies, and among those that were commonly applied the authors recommended the use of Ferrans and colleagues’ revised model to standardize HRQoL terminology and improve comparability between studies. In light of the growing interest in global health and adaptation of PRO instruments across populations and health conditions, potential next steps for this research could involve the application and cross-cultural validation of this model across geographical areas and health conditions for which HRQoL has not yet been well assessed.

Johnston et al. [8] provides an overview of five summary approaches for presenting pooled PRO estimates when conducting meta-analysis and pooling data across different HRQoL instruments that measure a common construct. A proposed next step for this research would be to evaluate the summary approaches that decision-makers such as clinicians, policy makers and patients find most useful and easy to understand.

The studies conducted by Irwin et al. [10] and Rajmil et al. [19] both underscore relatively new concepts in parent-proxy reporting, and lay the groundwork to advance this research across a broad range of pediatric-related health conditions as the samples in these studies were generally healthy participants.

Four studies pertained to the incorporation of HRQoL in economic evaluations, two of which described mapping disease-specific measures onto generic instruments. The structured review by Dakin [27] resulted in a database of studies mapping to the EQ-5D, and provided researchers with an efficient resource for identifying mapping algorithms. The author notes, however, that mapping should be considered secondary to direct measure given the additional errors and assumptions that this may introduce. Accordingly, a quality assessment of the mapping studies within the database could enhance this work, which in turn could potentially create opportunities for further research in cases where the quality is found to be sub-optimal.

Eight studies discussed various topics related to methodological issues and innovations in HRQoL measures. Lix et al. [39] evaluated the measurement equivalence of the SF-36 in a diverse sample of participants enrolled in the CaMos trial, and found that sex and race did not influence the conceptualization of a general measure of HRQoL. A proposed future direction for this research would be to replicate this work in other commonly used generic measures for which measurement equivalence is yet to be established in comparably diverse populations. Hutchings et al. [51] and Coste et al. [52] each assessed aspects of non-response bias on HRQoL estimates and found that non-response was associated with specific socio-demographic characteristics such as age and education level, and had an impact on the validity of the HRQoL estimates [52]. While the use of missing value methods such as multiple imputation as recommended by Coste [52] has clear implications for future studies, it would be interesting to see the effect of applying these methods on prior studies for which this consideration was not taken into account. Turner et al. [57] and Kuspiner et al. [58] aimed to determine the extent to which generic measures included important domains relevant to depression and MS, respectively. Though their results showed that none of the generic measures covered all domains deemed to be important by their study samples, they recommended the use of the SF-12 new algorithm for depression and SF-6D for MS as these were found to be the most comprehensive measures among those currently available. Wilson et al. [55] assessed the validity of applying population norms compared to retrospective analysis of pre-condition health among those affected by acute injury, and found that retrospective evaluation was a less biased measure of pre-injury health for those fully recovered at one-year follow-up. Khadka et al. [61] examined the characteristics of functional ratings scales in a sample of adult participants on a surgical waiting list, and found that items with simple and uniform question formats and four or five labeled categories demonstrated functionality, hierarchical ordering and good coverage of the latent trait under measurement. Krabbe and Forkman [63] assessed whether frequency or intensity scales should be employed as verbal anchors in PRO measures in a sample of participants with depressive disorder. Their results showed that both types of scales could be applied as verbal anchors, though they cautioned against using more than four adjacent terms as this may exceed the capacity for respondents to reasonably distinguish between categories. Given that the majority of these studies were specific to a particular health condition, reasonable next steps include the expansion of this research across other health conditions, and as noted by Kuspinar et al., [58] further developing condition-specific bolt-on items to generic utility measures and constructing utility measures containing only disease specific dimensions using the guidelines offered by Khadka et al. [61] and Krabbe and Forkman [63] as appropriate.

## Abbreviations

AQoL: Assessment of Quality of Life instrument
CaMos: Canadian Multi-centre Osteoporosis Study
EORTC QLQ-C30: European Organization for Research and Treatment of Cancer Quality of Life Questionnaire Core 30
EQ-5D: EuroQol five dimensions scale
EQ-5D-3 L: EuroQol five dimensions scale, 3 levels
EQ-5D VAS: EuroQol five dimensions scale Visual Analog Scale
HQLO: Health and Quality of Life Outcomes (journal)
HRQoL: Health Related Quality of Life
HUI2: Health-Utility Index-2
HUI3: Health-Utility Index-3
ICC: Intra-Class Correlation
KIDSCREEN: Screening for and promotion of health-related quality of life in children and adolescents
MID: Minimal Important Difference
NHS: National Health Service
NICE: National Institute for Health and Care Excellence
PRO: Patient-Reported Outcome
PROMIS: Patient-Reported Outcomes Measurement Information System
QoL: Quality of Life
RoM: Ratio of Means
SF-36: Medical Outcomes Study 36-item Short Form Health Survey
SF-12 new algorithm: 12-item Short Form Health Survey, new algorithm
SGRQ: St George Respiratory Questionnaire
SMD: Standard Mean Difference
WHODAS 2.0: World Health Organization Disability Assessment Schedule
